# Prenatal phthalate exposure and neurodevelopmental differences in twins at 2 years of age

**DOI:** 10.1186/s12889-024-17946-8

**Published:** 2024-02-20

**Authors:** Han Xiao, Liqin Hu, Tingting Tang, Jufang Zhong, Qiao Xu, Xiaonan Cai, Feiyan Xiang, Pan Yang, Hong Mei, Aifen Zhou

**Affiliations:** 1 Institute of Maternal and Child Health, Wuhan Children’s Hospital (Wuhan Maternal and Child Healthcare Hospital), Tongji Medical College, Huazhong University of Science and Technology, Wuhan, Hubei PR China; 2 Operating Room, Wuhan Children’s Hospital (Wuhan Maternal and Child Healthcare Hospital), Tongji Medical College, Huazhong University of Science and Technology, Wuhan, Hubei PR China; 3 Department of Obstetrics, Wuhan Children’s Hospital (Wuhan Maternal and Child Health Care Hospital), Tongji Medical College, Huazhong University of Science and Technology, Wuhan, China; 4 Delivery Room, Wuhan Children’s Hospital (Wuhan Maternal and Child Healthcare Hospital), Tongji Medical College, Huazhong University of Science and Technology, Wuhan, Hubei PR China; 5 Department of Public Health and Preventive Medicine, School of Medicine, Jinan University, 510632 Guangzhou, Guangdong PR China; 6 Guangdong Key Laboratory of Environmental Pollution and Health, Jinan University, 510632 Guangzhou, Guangdong PR China

**Keywords:** Phthalates, Neurodevelopmental differences, Twins, Urine

## Abstract

**Background:**

Previous studies of singletons evaluating prenatal phthalate exposure and early neurodevelopment reported mixed results and the associations could be biased by parental, obstetrical, and genetic factors.

**Methods:**

A co-twin control design was employed to test whether prenatal phthalate exposure was associated with children’s neurocognitive development. We collected information from 97 mother-twin pairs enrolled in the Wuhan Twin Birth Cohort between March 2016 and October 2018. Fourteen phthalate metabolites were measured in maternal urine collected at each trimester. Neurodevelopmental differences in twins at the age of two were examined as the outcome of interest. Multiple informant model was used to examine the covariate-adjusted associations of prenatal phthalate exposure with mental development index (MDI) and psychomotor development index (PDI) scores assessed at 2 years of age based on Bayley Scales of Infant Development (Second Edition). This model also helps to identify the exposure window of susceptibility.

**Results:**

Maternal urinary levels of mono-2-ethyl-5-oxohexyl phthalate (MEOHP) (β = 1.91, 95% CI: 0.43, 3.39), mono (2-ethyl-5-hydroxyhexyl) phthalate (MEHHP) (β = 1.56, 95% CI: 0.33, 2.79), and the sum of di-(2-ethylhexyl) phthalate metabolites (∑DEHP) (β = 1.85, 95% CI: 0.39, 3.31) during the first trimester showed the strongest and significant positive associations with intra-twin MDI difference. When stratified with twin chorionicity, the positive associations of monoethyl phthalate (MEP), monoisobutyl phthalate (MiBP), mono-n-butyl phthalate (MBP), monobenzyl phthalate (MBzP), individual DEHP metabolites, and ∑DEHP exposure during pregnancy with intra-twin neurodevelopmental differences were more significant in monochorionic diamniotic (MCDA) twins than those in dichorionic diamniotic (DCDA) twins.

**Conclusions:**

Neurodevelopmental differences in MCDA twins were strongly associated with prenatal phthalate exposure. Our findings warrant further confirmation in longitudinal studies with larger sample sizes.

**Supplementary Information:**

The online version contains supplementary material available at 10.1186/s12889-024-17946-8.

## Background

Phthalates are a class of synthetic chemicals widely used in the manufacture of plastics and other consumer products [1]. Because phthalates are not chemically bound to the products, they can easily leach into environmental medium [2], food [3], and drinking water [4], resulting in ubiquitous phthalate exposure in humans. Once absorbed, they are rapidly metabolized in the human body and principally excreted via urine. Phthalate metabolites have been frequently detected in urine samples from different population groups, including pregnant women [5–7].

Fetal exposure to phthalates may occur through their ability to cross the placenta [8]. Exposure to a low dose of di-(2-ethylhexyl) phthalate (DEHP) can also affect cerebrovascular function and increase the permeability of the blood-brain barrier [9]. Numerous food-monitoring studies have reported high concentrations of phthalates in various foods. Fetuses are more susceptible to environmental toxicants due to their insufficient detoxification ability and large amount of nutrition requirements for the rapid growth and brain development [10]. Toxicological studies have shown that phthalate exposure can disrupt the neuroendocrine systems (e.g., estrogenic signaling and metabolism), impair neuronal proliferation, differentiation and maturation, and have adverse effects on offspring’s neurodevelopment [11–12]. Increasing concerns have emerged over the adverse effects of prenatal phthalate exposure on the neurodevelopment of children. This is particularly significant due to the heightened vulnerability of children during the in-utero period to the influence of environmental toxicants [13].

A growing body of evidence has indicated that prenatal phthalate exposure is associated with a wide range of cognitive, social, behavioral, and emotional problems in preschool and school-aged children [14]. However, studies focused on children under the age of 3 years, which is considered one of the most vulnerable periods of development, are limited and report mixed results in terms of specific phthalates and neurocognitive performances. It is therefore an urgent need to clarify the relationships between phthalate exposure during pregnancy and early child development. Currently, in utero phthalate exposure assessment in the majority of previous studies was limited to a single spot urine sampled during the second or third trimester [6, 15–18], although chemical exposure at different time windows may induce differential effects on human’s central nervous system [19]. Two studies of singletons have examined the associations between trimester-specific phthalate exposure and child neurodevelopment [20–21], whereas these results may be biased by parental, obstetrical, and genetic factors.

Compared to singleton pregnancies, monochorionic diamniotic (MCDA) twins are at an increased risk for serious birth complications since the two fetal circulation systems are connected [22]. The imbalance of intertwin transfusion leads to unique hemodynamic manifestations for each twin, which may further increase their susceptibility to environmental exposures [23]. Twins account for 2–4% of newborns globally [24]. Despite similar genetic background, twins have discordant neurodevelopmental outcomes at a significant rate [25, 26]. Increasing evidence has suggested that the characteristics of the placenta, such as placental share, cord insertion site and placental morphology, vary between two twin fetuses including MCDA and dichorionic diamniotic (DCDA) twins [27, 28]. We speculate that twin pairs do not necessarily share a common in utero environment, and this hypothesis is supported by multiple twin studies showing that two fetuses within a pair can be subject to differential intrauterine exposure to chemicals such as bisphenols and air pollution [29–31]. MCDA twins have a same sex and share a same genetic structure and a single placenta; their phenotypic differences are largely attributed to different environmental exposures. Therefore, within-pair comparisons among twins, particularly those in MCDA twins, offer a unique opportunity to examine the relationships between prenatal phthalate exposure and neurodevelopment differences in children when controlling for key confounders shared within a twin pair, including parental, obstetrical, and genetic factors.

In the present study, we determined phthalate metabolite concentrations in maternal urine samples collected at each trimester, and further examined the relationships between trimester-specific phthalate exposure and intra-twin differences in neurodevelopment at 2 years of age and evaluated critical windows of susceptibility.

## Methods

### Study design and participants

The information used was collected from an ongoing twin birth cohort in Wuhan, China (Wuhan Twin Birth cohort, WTBC). Pregnant women were recruited from their first prenatal visit at Wuhan Children’s Hospital between March 2016 and October 2018. Participant recruitment, eligibility, and follow-up procedures were described elsewhere [32]. In brief, we enrolled women with twin pregnancies who were < 16 weeks of gestation, aged 18 years or older, living in Wuhan city, and planning to take prenatal care and deliver in the study hospital. Of the 432 initially enrolled participants, 286 completed a baseline questionnaire that included the information on pregnancy characteristics, socioeconomic levels, lifestyles, and disease history. After excluding twins without paired neurodevelopmental assessment at 2 years of age, our final analysis consisted of 97 mother-twin pairs (Supplementary Fig. S1).

### Maternal phthalate exposure assessment

Women pregnant with twins provided one-spot first morning urine sample in polypropylene containers at each of three prenatal visits. Of the study participants, 97, 97, and 94 women provided urine samples at their first, second, and third trimesters (median = 13, 24, and 30 weeks of gestation), respectively. After collection, the urine samples were aliquoted into polypropylene tubes and stored at − 20 °C until analysis.

Phthalate metabolites were analyzed using ultra-performance liquid chromatography-tandem mass spectrometry (UPLC-MS/MS, Applied Biosystem/AB SCIEX 5500 Triple Quadrupole) with a negative electrospray ionization mode. The detail of pretreatment, separation, and measurement procedures was described previously [33]. We focused on 8 phthalate metabolites that had ≥ 85% of concentrations above the limits of detection (LODs), including monoethyl phthalate (MEP), monoisobutyl phthalate (MiBP), mono-n-butyl phthalate (MBP), monobenzyl phthalate (MBzP), four metabolites of DEHP [mono-2-ethylhexyl phthalate (MEHP), mono (mono-2-ethyl-5-oxohexyl phthalate (MEOHP), 2-ethyl-5-hydroxyhexyl) phthalate (MEHHP), mono-2-ethyl-5-carboxypentyl phthalate (MECPP)]. LODs for phthalate metabolites ranged from 0.02 to 0.11 ng/mL, and chemical concentrations below the LOD were substituted with LOD/√2. MEHP, MEOHP, MEHHP, and MECPP were produced by the metabolism of DEHP, thus we calculated a molar sum of the four metabolites (∑DEHP, expressed in micromoles per liter) for estimating the total exposure concentration of DEHP. The quality accuracy/quality control (QA/QC) procedures were performed by regular analysis of procedure blanks, matrix-matched calibration standards, and surrogate standards with high and low concentrations in urine matrix. After subtracting background contamination, the mean recovery of matrix spiked standards ranged from 90.0−111.4% with relative standard deviations (RSDs) of 7.6–19.5%.

We measured specific-gravity (SG) levels in maternal urine samples using a digital refractometer (Atago PAL-10 S, Tokyo, Japan), and used SG to correct for urine dilution according to the following equation: Pc = P × [(SG_m_-1) / (SG-1)]. P is the detected phthalate metabolite concentrations, SG_m_ is the median SG for the urine samples from our participants, and SG is specific gravity of individual urine samples.

### Neurodevelopmental assessment

We invited mother and their infants to return to Wuhan Children’s Hospital at approximately 2 years of age. At this period, trained pediatricians administered the Chinese revision of Bayley Scales of Infant Development (BSID-CR) to assess the cognitive and motor development of children. BSID-CR has been a valid screening scale for neurodevelopmental assessment in Chinese children aged 2–30 months [20]. Similar to Bayley Scales of Infant Development-II (BSID-II), BSID-CR also generates two indices: mental development index (MDI) and psychomotor development index (PDI). The former assesses children’s cognitive, language, and social skills, and the latter assesses gross- and fine-motor skills. Higher MDI and PDI scores indicate better neurocognitive development.

### Data Collection

During the first prenatal visit, a structured questionnaire administered by trained staffs was provided to mothers for information collection, including maternal age, prepregnancy weight and height, education, the use of assisted reproductive technology (ART), self-reported cosmetic use, and second-hand smoke exposure in pregnancy. We extracted medical information, such as gestational age, twin birth weight, twin chorionicity, and sex of twin fetuses by hospital registries after delivery. In this study, intra-twin differences in neurodevelopment were the outcome of interest, which were estimated by subtracting the lower MDI or PDI score from the higher MDI or PDI score, respectively. We also calculated the covariate of birth weight discordance using the following formula: 100% × (larger twin-smaller twin)/larger twin [34], since birth weight discordance ≥ 20% was significantly associated with long-term neurodevelopmental differences [35].

### Statistical analyses

Descriptive statistics were performed for participants’ basic characteristics. Chi-square tests and independent t‑tests were used to compare differences in the characteristics between participants we followed (*n* = 97) and those lost to follow-up (*n* = 189). For the study participants, we calculated Spearman correlation coefficients to assess the correlations between SG-corrected phthalate metabolites by trimesters. Random intercept linear mixed models were applied to evaluate the within-subject variability of urinary phthalate metabolites across the three trimesters, with results expressed as intraclass correlation coefficients (ICCs) and 95% confidant intervals (CIs). ICCs > 0.75 indicated low variability, 0.40–0.75 indicated moderate variability, and < 0.40 indicated high variability.

We used multiple informant models to examine the associations between phthalate metabolites and neurodevelopmental differences in twins at the age of two [36]. Concentrations of phthalate metabolites were modeled as both continuous (ln-transformed) and categorical (tertiles) variables. Linear trend tests by tertiles of phthalate metabolites were estimated by modeling the median value of each tertile, with lowest tertile as the reference. Multiple informant models treat different exposure windows (trimesters) as informants and jointly estimate intra-twin neurodevelopmental differences in relation to individual chemical biomarker concentrations in each trimester. This method can be used to test the differences between trimester-specific exposure and neurodevelopment, and a type 3 *P* value < 0.10 indicated that the associations differ across trimesters.

To eliminate influences of obstetrical, parental, and genetic factors on child neurodevelopment, we further conducted stratified analysis by twin chorionicity. Because identical twins (MCDA twins) have a same sex and share a same genetic structure and a single placenta, their intra-twin comparison analyses can help to determine the adverse health effects of environmental exposure. In detail, the associations between ln-transformed urinary phthalate metabolites and neurodevelopmental differences were separately examined in MCDA and DCDA twins. We did interaction test between individual phthalate metabolites in each trimester and twin chorionicity using the Wald statistic, with a *P*_interaction_ value < 0.10 considered significant [37].

We used a directed acyclic graph to determine potential covariates in the adjusted models (Supplementary Fig. S2); the variables included in the graph were selected *a priori* based on previous publications [20, 21, 34]. The minimal sufficient covariates included maternal age (continuous), pre-pregnancy BMI (continuous), education (high school or below degree vs. college or above degree), chorionicity (MCDA vs. DCDA), and sex types of twin fetuses (male-male vs. female-female vs. male-female) in the multivariate models.

We carried out additional sensitivity analyses to assess the robustness of our results. To address the issue of multiple comparisons, we employed the Benjamini-Hochberg False Discovery Rate correction. To avoid over- or undercorrection, we first analyzed the associations between phthalate metabolites and neurodevelopmental differences in twins without adjustments for any confounders. We additionally adjusted for other potential confounders such as self-reported cosmetic use and secondhand smoke exposure during pregnancy in the statistical models. Children conceived via ART were excluded from the analysis, as previous studies have suggested that assisted conception had a negative impact on child neurodevelopment [38, 39]. Finally, twins that had a discordant birth weight were excluded since intra-twin birth weight discordance of ≥ 20% has been shown to predict neurodevelopmental outcomes in children throughout childhood [35]. SAS version 9.4 (SAS Institute, Inc., Cary, NC, USA) was used to conduct the statistical analyses. A two-tailed *P* < 0.05 indicated statistical significance.

## Results

### Cohort characteristics

The characteristics of the study participants are displayed in Table 1. Maternal participants had a mean (± SD) age and prepregnancy body mass index (BMI) of 30.7 ± 3.9 years and 21.9 ± 3.1 kg/m^2^, respectively. The majority of mothers had at most a high school degree (79.4%), were not exposed to secondhand smoke (72.7%) and did not use cosmetics during pregnancy (51.9%). Infants were an average of 36.7 ± 1.4 weeks gestational age at birth. Approximately 68.0% of them were DCDA twins, 13.4% were discordant for birthweight, and 29.9% were conceived via ART. The mean (± SD) intra-twin differences in Bayley scores were 10.8 ± 7.8 points for MDI and 11.4 ± 11.2 points for PDI.

**Table 1:** Demographic characteristics of the pregnant women and their fetuses in this study

Characteristics	Mean ± SD or n (%)		
	Overall (*n* = 97)	MCDA (*n* = 31)	DCDA (*n* = 66)
**Mother**			
Maternal age (years)	30.7 ± 3.9	30.0 ± 4.4	31.1 ± 3.6
Pre-pregnancy BMI (kg/m^2^)	21.9 ± 3.1	21.3 ± 2.9	22.2 ± 3.2
Maternal education, n (%)			
High school or below degree	77 (79.4)	26 (83.9)	51 (77.3)
College or above degree	20 (20.6)	5 (16.1)	15 (22.7)
Secondhand smoke exposure in pregnancy, n (%)			
NO	70 (72.2)	22 (71.0)	48 (72.7)
YES	27 (27.8)	9 (29.0)	18 (27.3)
Self-reported cosmetic use, n (%)			
NO	50 (51.9)	10 (32.3)	40 (60.6)
YES	47 (48.5)	21 (67.7)	26 (39.4)
**Twins**			
Gestational age (weeks)	36.7 ± 1.4	36.7 ± 1.6	36.7 ± 1.2
Birthweight discordance, n (%)			
≤ 20%	84 (86.6)	27 (87.1)	57 (86.4)
> 20%	13 (13.4)	4 (12.9)	9 (13.6)
Sex types of twin fetuses, n (%)			
Male-male	37 (38.2)	17 (54.8)	20 (30.3)
Female-female	33 (34.0)	14 (45.2)	19 (28.8)
Male-female	27 (27.8)	0 (0)	27 (40.9)
The use of ART, n (%)			
NO	68 (70.1)	30 (96.8)	38 (57.6)
YES	29 (29.9)	1 (3.2)	28 (42.4)
Intra-twin MDI difference	10.8 ± 7.8	9.6 ± 6.1	11.3 ± 8.5
Intra-twin PDI difference	11.4 ± 11.2	10.0 ± 11.2	12.1 ± 11.2

Abbreviations: SD, standard deviation; BMI, body mass index; ART, artificial reproductive technology; MDI, mental development index; PDI, psychomotor development index

Supplementary Table S1 displays baseline characteristics of participants who followed (*n* = 97) and those lost to follow-up (*n* = 189), and no significant differences were observed between the two groups with respect to the important characteristics of pregnant women and their twin fetuses. Also, we compared the concentration distribution of standardized phthalate metabolites in these two groups, and similar concentrations were found for almost all of metabolites, with the exception of second-trimester MEHP and third-trimester MEP (Supplementary Table S2).

### Urinary phthalate metabolite concentrations during pregnancy

**Fig. 1:**
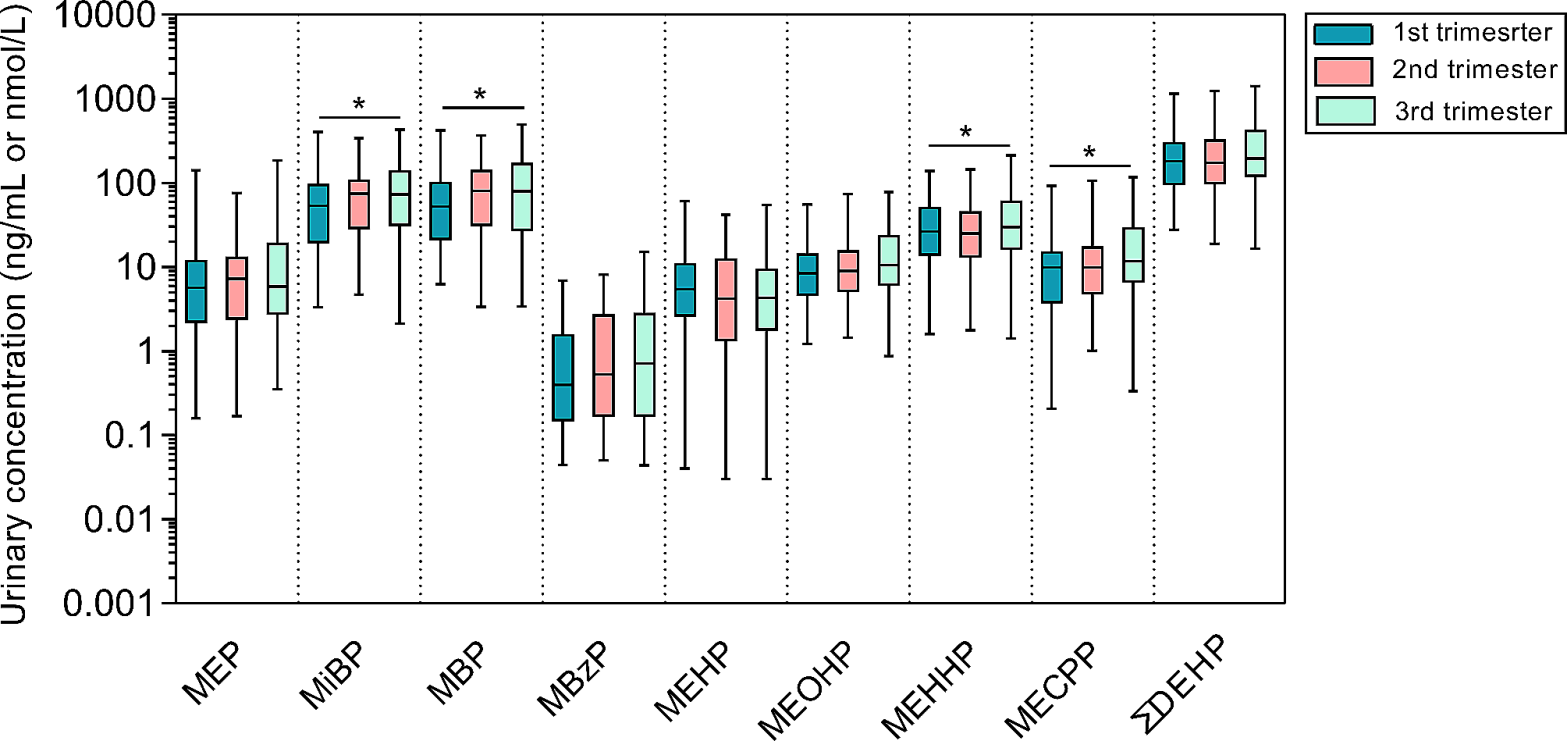
Distributions of concentrations of urinary phthalate metabolites across entire pregnancy. The bottom, the line inside, and top of the box showed the first, second, and third quartiles. The whiskers were 2.5% and 97.5% values

The distribution of SG-corrected phthalate metabolite concentrations across three trimesters are provided in Table 2; Fig. 1. MiBP and MBP had the highest phthalate concentration, followed by DEHP metabolites and MEP, and the lowest concentration was determined for MBzP. Except for MEHP, concentrations of individual metabolites showed increasing trends with pregnancy progressed, and significant differences in MiBP, MBP, MEHHP, and MECPP levels were observed between the first and third trimesters (Fig. 1).

**Table 2:** The summary of specific gravity-corrected urinary phthalate concentrations in pregnant women across three trimesters (*n* = 97)

Phthalate metabolites (ng/mL)	>LOD (%)	Median	Geometric mean (95% CI)			
			Entire pregnancy	1st trimester	2nd trimester	3rd trimester
MEP	97.9–99.0	5.88	5.98 (5.02, 7.12)	5.19 (3.88, 6.96)	5.69 (4.25, 7.62)	7.27 (5.23, 10.1)
MiBP	98.9–100	63.3	51.0 (43.9, 59.3)	42.3 (32.8, 55.8)	53.2 (42.6, 66.2)	59.3 (44.3, 78.6)
MBP	98.9–100	68.3	56.2 (48.9, 64.6)	46.5 (36.8, 58.6)	59.2 (46.8, 74.2)	64.9 (49.3, 84.4)
MBzP	84.5–88.7	0.53	0.59 (0.49, 0.70)	0.48 (0.38, 0.64)	0.62 (0.45, 0.82)	0.68 (0.43, 0.96)
MEHP	84.5–93.8	4.72	3.46 (2.80, 4.27)	4.41 (3.28, 6.04)	2.65 (1.74, 4.02)	3.55 (2.43, 5.06)
MEOHP	98.9–100	9.25	9.28 (8.28, 10.4)	8.17 (6.68, 10.0)	8.96 (7.53, 10.2)	10.9 (8.83, 13.6)
MEHHP	98.9–100	27.3	24.7 (21.8, 28.1)	23.8 (18.8, 30.0)	23.1 (19.1, 28.2)	27.6 (21.3, 35.2)
MECPP	98.9–100	10.6	9.38 (8.13, 10.8)	7.56 (5.88, 9.73)	9.40 (7.60, 11.2)	11.6 (8.83, 15.4)
∑DEHP (nmol/L)	–	186	181 (162, 202)	175 (147, 208)	166 (139, 200)	206 (166, 256)

Abbreviations: LOD, limit of detection

### Variability and correlations of phthalate metabolites across trimesters

The variability and correlations of SG-corrected phthalate metabolites throughout pregnancy are represented in Table 3. MiBP (ICC = 0.49) and MBP (ICC = 0.52) concentrations showed moderate variability, while the remaining seven metabolites showed high variability (ICCs < 0.45). Spearman correlation coefficients of urinary phthalate metabolites across three trimesters suggested that short time intervals (second trimester vs. first or third trimester) correlated better than long time interval (first trimester vs. third trimester).

**Table 3:** The overall intraclass correlation coefficient (ICC) and Spearmen’s correlation coefficient for phthalate metabolites across trimesters

Phthalate metabolites (ng/mL)	ICC (95% CI)	Spearmen’s correlation coefficient		
		1st vs. 2nd trimester	2nd vs. 3rd trimester	1st vs. 3rd trimester
MEP	0.25 (0.13, 0.39)	0.32 **	0.30 **	0.20 *
MiBP	0.49 (0.36, 0.60)	0.44 **	0.42 **	0.34 **
MBP	0.52 (0.40, 0.63)	0.63 **	0.59 **	0.36 **
MBzP	0.42 (0.30, 0.55)	0.43 **	0.53 **	0.34 **
MEHP	0.27 (0.14, 0.40)	0.23 *	0.34 **	0.14
MEOHP	0.30 (0.17, 0.43)	0.25 *	0.37 **	0.13
MEHHP	0.29 (0.16, 0.42)	0.16	0.27 **	0.15
MECPP	0.27 (0.14, 0.41)	0.28 **	0.40 **	0.24 *
∑DEHP (nmol/L)	0.20 (0.08, 0.34)	0.20 *	0.34 **	0.14

*P* < 0.05 (*) and *P* < 0.01 (**) represents significant correlation

### Associations between phthalate metabolites and Bayley scores

The trimester-specific associations of ln-transformed phthalate metabolites with intra-twin differences in Bayley scores are presented in Table 4. We found that ln-transformed levels of maternal urinary MEOHP (β = 1.91, 95% CI: 0.43, 3.39), MEHHP (β = 1.56, 95% CI: 0.33, 2.79), and ∑DEHP (β = 1.85, 95% CI: 0.39, 3.31) during the first trimester were associated with an increased intra-twin MDI difference, and these associations varied by trimesters (all of the heterogeneity *P*-values < 0.10). However, no significant associations were observed between phthalate exposure in each trimester and intra-twin PDI difference, and no evidence indicated that these insignificant associations differed across the window of exposure.

**Table 4:** Associations between ln-transformed urinary phthalate metabolites and intra-twin differences in neurodevelopment (*n* = 97) ^a^

Phthalate metabolites	1st trimester			2nd trimester			3rd trimester		P_tri–int_^b^
	β (95% CI)	*P*–value		β (95% CI)	*P*–value		β (95% CI)	*P*–value	
Intra-twin MDI difference									
MEP	–0.54 (–1.48, 0.40)	0.26		0.07 (–0.84, 0.98)	0.89		–0.50 (–1.23, 0.23)	0.18	0.48
MiBP	0.38 (–0.94, 1.69)	0.57		0.40 (–0.83, 1.62)	0.53		–0.86 (–2.35, 0.64)	0.26	0.31
MBP	–0.01 (–1.32, 1.29)	0.99		0.17 (–0.98, 1.32)	0.77		–0.71 (–2.01, 0.58)	0.28	0.42
MBzP	–0.29 (–1.41, 0.83)	0.62		0.14 (–0.70, 0.98)	0.74		0.08 (–0.87, 1.04)	0.86	0.87
MEHP	0.47 (–0.50, 1.45)	0.34		0.41 (–0.19, 1.01)	0.18		–0.42 (–1.51, 0.66)	0.44	0.46
MEOHP	**1.91 (0.43, 3.39)**	**0.01**		0.48 (–0.77, 1.73)	0.45		–0.63 (–2.03, 0.76)	0.37	**0.04**
MEHHP	**1.56 (0.33, 2.79)**	**0.01**		0.53 (–0.70, 1.75)	0.40		–0.48 (–1.63, 0.67)	0.41	**0.06**
MECPP	0.73 (–0.64, 2.11)	0.30		0.22 (–0.84, 1.29)	0.68		–0.23 (–1.23, 0.78)	0.66	0.61
∑DEHP	**1.85 (0.39, 3.31)**	**0.01**		0.57 (–0.69, 1.83)	0.37		–0.53 (–1.62, 0.56)	0.34	**0.06**
Intra-twin PDI difference									
MEP	0.16 (–1.54, 1.85)	0.86		–0.53 (–2.11, 1.05)	0.51		0.93 (–0.33, 2.18)	0.15	0.31
MiBP	–0.44 (–2.51, 1.62)	0.68		–1.33 (–3.28, 0.62)	0.18		0.73 (–1.79, 3.25)	0.57	0.38
MBP	0.23 (–1.86, 2.33)	0.83		–0.81 (–2.70, 1.08)	0.40		0.46 (–1.65, 2.58)	0.67	0.52
MBzP	–1.01 (–2.38, 0.36)	0.15		0.58 (–0.77, 1.93)	0.40		0.70 (–0.63, 2.04)	0.30	0.17
MEHP	0.89 (–0.19, 1.98)	0.11		0.18 (–0.86, 1.22)	0.74		0.39 (–0.75, 1.52)	0.50	0.41
MEOHP	–1.15 (–4.14, 1.84)	0.45		–0.04 (–2.50, 2.43)	0.98		1.70 (–0.81, 4.21)	0.19	0.43
MEHHP	–1.27 (–4.12, 1.58)	0.38		0.19 (–2.17, 2.55)	0.87		0.92 (–1.23, 3.07)	0.4	0.67
MECPP	–0.67 (–2.47, 1.13)	0.46		–0.70 (–2.59, 1.18)	0.46		0.47 (–1.23, 2.18)	0.59	0.60
∑DEHP	–1.38 (–4.32, 1.55)	0.36		0.02 (–2.32, 2.37)	0.98		1.10 (–0.85, 3.05)	0.27	0.53

Abbreviations: CI, confidence interval

^a^ Models are adjusted for maternal age, pre-pregnancy BMI, education, chorionicity, and sex types of twin fetuses

^b^ Score test of homogeneity of effect estimates across the three trimesters

Bold format indicates significant results

We also examined tertiles of phthalate metabolites and intra-twin differences in Bayley scores (Supplementary Fig. S3), and consistent results were observed when phthalate metabolite levels were modeled as continuous or tertile variables. Specifically, compared to the lowest tertiles, the highest tertiles of maternal urinary MEOHP (β = 4.10, 95% CI: 0.84, 7.36), MEHHP (β = 3.41, 95% CI: 0.37, 6.45), and ∑DEHP (β = 3.68, 95% CI: 0.52, 6.84) during the first trimester were associated with an increased intra-twin MDI difference. The associations between phthalate metabolites across three trimesters and intra-twin PDI difference were not significant.

### Analysis stratified by twin chorionicity

**Fig. 2:**
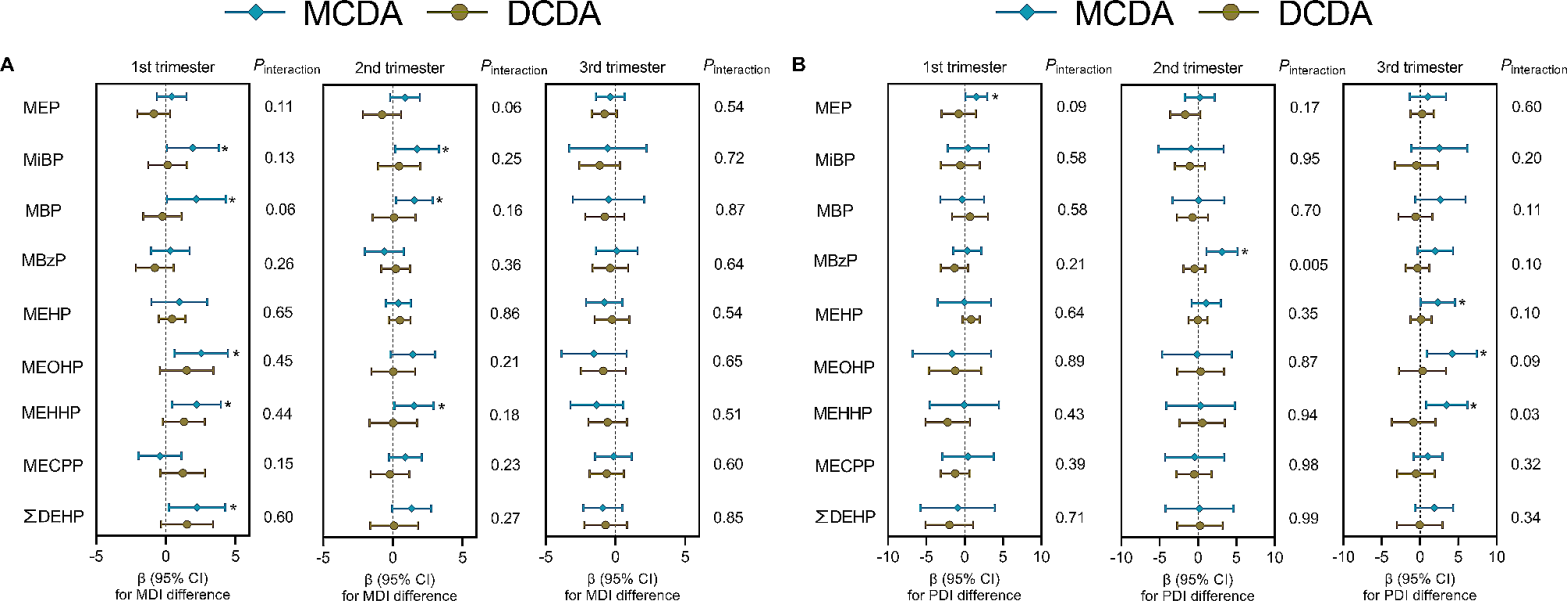
Associations between urinary phthalate metabolites and intra-twin MDI (**A**) and PDI (**B**) differences in monochorionic diamniotic (MCDA) and dichorionic diamniotic (DCDA) twins. Models are adjusted for maternal age, pre-pregnancy BMI, education, chorionicity, and sex types of twin fetuses. * *P* < 0.05

Figure 2 shows the associations between maternal urinary phthalate metabolites and intra-twin differences in Bayley scores by the strata of twin chorionicity. In MCDA twins, MiBP (β = 1.92; 95% CI: 0.03, 3.81 for the first trimester and β = 1.73; 95% CI: 0.16, 3.30 for the second trimester), MBP (β = 2.16; 95% CI: 0.01, 4.31 for the first trimester and β = 1.55; 95% CI: 0.23, 2.87 for the second trimester), individual DEHP metabolites (MEOHP: β = 2.54; 95% CI: 0.62, 4.46 for the first trimester; MEHHP: β = 2.21; 95% CI: 0.46, 3.96 for the first trimester and β = 1.53; 95% CI: 0.13, 2.93 for the second trimester), and ∑DEHP (β = 2.24; 95% CI: 0.23, 4.25 for the first trimester) exposure during pregnancy was associated with an increased intra-twin MDI difference. Additionally, MEP (β = 1.53; 95% CI: 0.10, 2.95 for the first trimester), MBzP (β = 3.14; 95% CI: 1.11, 5.16 for the second trimester), and individual DEHP (MEHP: β = 2.33; 95% CI: 0.08, 4.58, MEOHP: β = 4.20; 95% CI: 0.94, 7.45, and MEHHP: β = 3.48; 95% CI: 0.77, 6.19 for the third trimester) exposure was positively associated with an intra-twin PDI difference. We also observed evidence that some associations were stronger in MCDA twins than those in DCDA twins (*P*_interaction_ < 0.10).

### Sensitive analyses

In sensitivity analyses, the main results did not change appreciably after (a) without adjustment for any confounders (Supplementary Table S3), (b) additionally adjusting for other potential confounders, including self-reported cosmetic use and second-hand smoke exposure in pregnancy (Supplementary Table S4), (c) excluding 29 twin pairs conceived via ART (Supplementary Table S5), (d) excluding 13 twin pairs with a discordant birthweight (Supplementary Table S6), and (e) implementing the Benjamini-Hochberg correction to control for false discovery rate (Supplementary Table S7).

## Discussion

Using data from prospective cohort of twin pregnant women and their fetuses, we examined the associations of urinary phthalate metabolite concentrations at each trimester with intra-twin differences in Bayley scores at 2 years of age. We observed that maternal urinary MiBP, MBP, MEOHP, MEHHP, and ∑DEHP during the first and second trimesters were strongly associated with an intra-pair MDI difference in MCDA twins, implying unshared in utero exposure to phthalates between the MCDA twins who shared a single placenta. Our study identified early pregnancy as the vulnerable window.

### Exposure assessment

We compared the median concentrations of phthalate metabolites in urine from pregnant women throughout the world (shown in Supplementary Fig. S4). A similar exposure pattern was observed between this present work and previous studies of women pregnant with singletons in China [20, 40]. Moreover, the median concentrations of MBP, MEHP, MEOHP, MEHHP, and MECPP in our participants were comparable to those in pregnant women from France [41], the USA [6, 42], and Saudi Arabia [5], but higher than the results from Demark [43] and Canada [44]. However, urinary MEP was 10–40 times lower than those reported in studies from France [41], the USA [6], and Saudi Arabia [5]. The different exposure patterns for phthalates may be attributed to the variations in study population, sampling years, gestational age at sample collection, lifestyles, and dietary habits [45–47].

Phthalate metabolites in the present study exhibited an increasing trend in concentrations over pregnancy, except for MEHP. Significant differences in MiBP, MBP, MEHHP, and MECPP levels were observed between the first and third trimesters. The possible explanation is that mothers in late pregnancy may be subjected to more extensive phthalate exposure from dietary intake and indoor microenvironments. A previous study conducted on the general population has suggested that the consumption of ultra-processed foods, such as sandwiches, hamburgers, French fries, and other potato products, was associated with higher concentrations of urinary phthalate metabolites [3]. Pregnant women are exposed to higher levels of phthalates through their dietary intake because they require a larger amount of nutrition to ensure adequate growth and organ development for the fetus, particularly during late pregnancy [48]. Moreover, mothers in late pregnancy tend to spend more time indoors than in early pregnancy [49], and indoor air and dust are commonly considered significant sources of exposure to phthalates [50]. Anatomical, physiological and metabolic changes (e.g., enzyme activities, organ volumes and blood flows, and glomerular filtration rates) in pregnancy could be considered other possible reasons [51].

In our study, the ICCs for most phthalate metabolites (e.g., MiBP, MBP, and DEHP metabolites) were similar to those estimated in other studies with multiple spot urine samples collected from a single day to months [52–54], but the ICCs for MEP and MBzP were lower than the values reported from Belgium [52] and the USA [53, 54]. In general, most phthalate metabolite concentrations are of high variability across pregnancy, thus repeated measurements for urinary phthalate metabolites are warranted to reduce misclassification bias in exposure assessment.

### Phthalate metabolite concentrations and Bayley scores

The “first 1000 days” is widely recognized as the golden brain opportunity, because a great deal of the brain’s ultimate structure and capacity is shaped during this period. Failure to optimize brain development in early life can have long-term effects to education, job potential, and adult mental health [55]. Previous studies of singletons evaluating prenatal phthalate exposure and children’s Bayley scores during the “first 1000 days” showed mixed results. For instance, Qian et al. (2019) report a negative association between an average concentration of MBP in different three trimesters and PDI score in 2-year-old children. The Mothers and Children’s Environmental Health (MOCEH) study suggest that maternal urinary MEHHP and MEOHP are inversely associated with MDI and PDI scores in children aged 6 months [16], and this study team also observe a reduction of MDI and PDI scores in association with prenatal MEP exposure [15]. However, several studies indicate null associations of maternal urinary phthalate metabolites with MDI and PDI scores [6, 17, 18].

The outcome variables in this present study are intra-twin MDI and PDI differences, which reflect the relative differences in neurocognitive performances, making it difficult to directly compare this study with previous studies of singletons. In our stratified analysis, the positive associations of maternal urinary MEP, MiBP, MBP, MBzP, individual DEHP metabolites, and ∑DEHP with intra-pair difference were only significant in MCDA twins. MCDA twins share a single placenta and can have unequal placental sharing [27, 56], and phthalates have been proved to across the human placenta [9]. Given a shared genetic structure, a discordant neurodevelopment between two MCDA twins are largely attributed to the different exposure in utero. A previous singleton study has reported a significant decrease in MDI score of children exposed to MBP, MEOHP, and MEHHP [16], thus we speculate that the intra-pair MDI difference in MCDA twins may be attributed to differential phthalate exposure in utero. In our previous study, we have determined maternal and cord plasma concentrations of poly- and perfluoroalkyl substances (PFAS), and indeed observed a significant exposure difference for twins within a pair (*P* < 0.001) [57]. The reason why two twins have discordant chemical exposure may be due to the presence of vascular anastomoses on the placental surface that connect the 2 fetal circulation systems. Anastomoses can be of 3 types: arterioarterial (AA), venovenous (VV), and arteriovenous (AV), with AV anastomoses having unidirectional blood flow [58]. Because of their unidirectional nature, AV anastomoses can create a transfusion imbalance and further lead to some unique complications in MCDA twins, such as birthweight discordance and neurodevelopment difference [59]. Although approximately 2-point difference in MDI score may result in a small effect for individuals, it can produce a profound societal impact when extended to the entire population.

### The critical window of susceptibility

In our study, first-trimester DEHP metabolites showed the strongest and significant associations with intra-pair neurodevelopmental differences in overall twin pairs, indicating that early pregnancy is the critical exposure window of susceptibility. Our findings are concordant with the ELEMENT cohort study which reported negative associations between first-trimester phthalate exposure and children’s motor, cognitive and memory abilities [21]. Moreover, previous reviews based on evidence from humans and animal models indicate that the developmental processes of the nervous system begin early in embryogenesis, and fetuses are highly sensitive to the risk factors for brain development [60].

### Biological mechanisms

Phthalates are endocrine-disruptive chemicals that may exert the neurodevelopmental toxicity via the interaction with neuroendocrine systems. Human evidence and animal studies have found that parental di-isobutyl phthalate (DiBP), di-n-butyl phthalate (DBP), and DEHP exposure can disrupt the regulation and homeostasis of sex hormones and thyroid hormones in offspring [61–65]. Neuroendocrine hormones are crucial for the developmental processes (e.g., migration, synaptogenesis, and myelination) of the nervous system, thus the disruption of in utero hormone environment by phthalates may increase the risk of neurodevelopmental defects during fetal life and childhood [66, 67]. DBP and DEHP have also been shown to disturb the expressions of dopamine receptor, tyrosine hydroxylase enzyme, and brain-derived neurotrophic factor (BDNF), and adversely affect neurodevelopment [68–70].

### Strength and limitation

Our study adds to the literature of phthalate exposure with intra-twin differences in Bayley scores at age of two, to our knowledge, is the first study to report significant trimester-specific associations between maternal urinary phthalate metabolites and intra-twin MDI difference, providing new insight to assess the risk of phthalate exposure on child neurodevelopment.

However, several limitations exist in this study. First, although we controlled for some key confounders, such as self-reported cosmetic use, twin chorionicity, and growth discordance, the unmeasured factors may bias our findings. One plausible source of residual confounding is by other correlated neurotoxicants (e.g., lead, phenolic substances), since real-life entails simultaneous exposure to multiple chemicals. Second, the intra-twin MDI differences may be attributed to postnatal exposure as well, for example, the smaller twin may have been in the neonatal intensive care unit (ICU) and exposed to plastic tubing. Third, approximately two third of baseline population was lost for 24-month follow-up, thus selection bias may exist. However, we observed no notable differences in baseline characteristics between participants we followed and those lost to follow-up. Urinary phthalate metabolite levels were also comparable between these two groups. It should be noted that identifying differences in exposure is crucial for understanding potential mechanisms underlying phenotypic discordance in twin pairs. This is because maternal phthalate exposure only reflects the total exposure of both twin fetuses. Further study is needed to associate individual phthalate exposure (fetal cord blood or meconium) with neurodevelopmental differences in twins. Last, our results should be interpreted with caution due to a small sample size.

## Conclusions

The present study of twins indicated that first-trimester DEHP exposure was associated with an increased intra-twin MDI difference in all of twin pairs. Compared to DCDA twins, the associations between prenatal phthalate exposure and intra-twin neurodevelopmental differences were stronger in MCDA twins. Further, larger prospective studies performed on MCDA twins are needed to confirm our findings and uncover the potential mechanisms involved placental characteristics.

### Electronic supplementary material

Below is the link to the electronic supplementary material.

Supplementary Material 1

Supplementary Material 2

## Data Availability

The datasets used and/or analysed during the current study available from the corresponding author on reasonable request.

## Abbreviations

MDI: Mental development index
PDI: Psychomotor development index
BSID-CR: Chinese revision of Bayley Scales of Infant Development
MCDA: Monochorionic diamniotic
DCDA: Dichorionic diamniotic
UPLC-MS/MS: Ultra-performance liquid chromatography-tandem mass spectrometry
LOD: Limits of detection
MEP: Monoethyl phthalate
MiBP: Monoisobutyl phthalate
MBP: Mono-n-butyl phthalate
MBzP: Monobenzyl phthalate
MEHP: Mono-2-ethylhexyl phthalate
MEOHP: Mono (mono-2-ethyl-5-oxohexyl phthalate
MEHHP: 2-ethyl-5-hydroxyhexyl) phthalate
MECPP: Mono-2-ethyl-5-carboxypentyl phthalate
DEHP: Di-(2-ethylhexyl) phthalate
BMI: Body mass index
ART: Artificial reproductive technology
