# Supplementary material for: Prenatal phthalate exposure and neurodevelopmental differences in twins at 2 years of age
Source: BMC Public Health. 2024 Feb 20;24:533. doi: 10.1186/s12889-024-17946-8 (PMC10880363; doi:10.1186/s12889-024-17946-8)
Supplement: Supplementary file 2 — Supplementary Material 2 [file 12889_2024_17946_MOESM2_ESM.docx]

**Prenatal phthalate exposure and** **neurodevelopmental differences in twins at 2 years of age**

Han Xiao ^a,1^, Liqin Hu ^a,1^, Tingting Tang ^b^, Jufang Zhong ^c^, Qiao Xu ^d^, Xiaonan Cai ^a^, Feiyan Xiang ^a^, Pan Yang ^e, f^, Hong Mei ^a,^ *, Aifen Zhou ^a,^ *

^a^ Institute of Maternal and Child Health, Wuhan Children’s Hospital (Wuhan Maternal and Child Healthcare Hospital), Tongji Medical College, Huazhong University of Science and Technology, Wuhan, Hubei, PR China

^b^ Operating Room, Wuhan Children’s Hospital (Wuhan Maternal and Child Healthcare Hospital), Tongji Medical College, Huazhong University of Science and Technology, Wuhan, Hubei, PR China

^c^ Department of Obstetrics, Wuhan Children's Hospital (Wuhan Maternal and Child Health Care Hospital), Tongji Medical College, Huazhong University of Science and Technology, Wuhan, China.

^d^ Delivery Room, Wuhan Children’s Hospital (Wuhan Maternal and Child Healthcare Hospital), Tongji Medical College, Huazhong University of Science and Technology, Wuhan, Hubei, PR China

^e^ Department of Public Health and Preventive Medicine, School of Medicine, Jinan University, Guangzhou 510632, Guangdong, PR China.

^f^ Guangdong Key Laboratory of Environmental Pollution and Health, Jinan University, Guangzhou 510632, Guangdong, PR China.

* Corresponding author.

E-mail: [april1972@163.com](mailto:april1972@163.com); hongmei2017@hotmail.com.

^1^ Han Xiao and Liqin Hu contributed equally to the article.

**
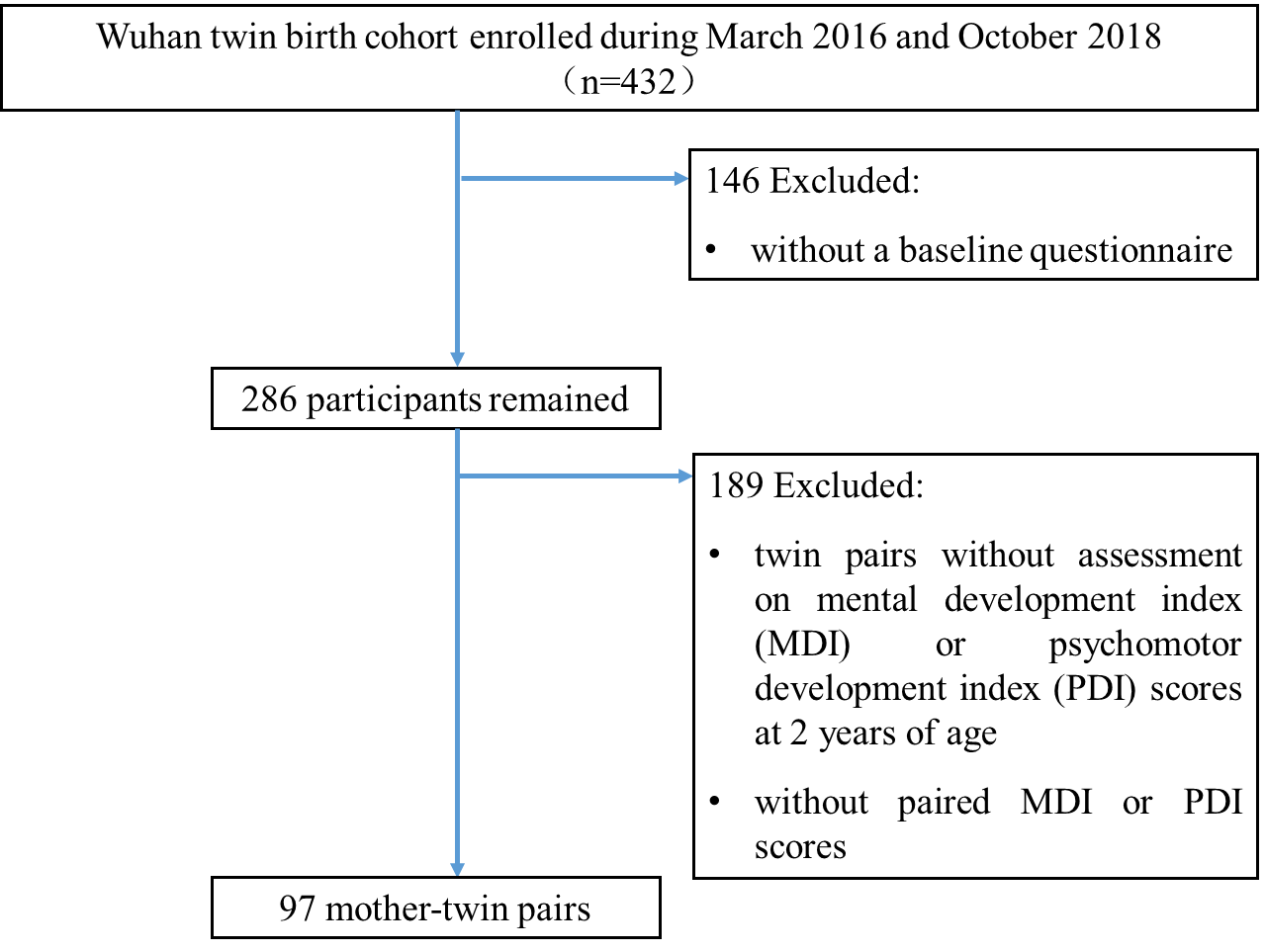
**

**Fig S1. The detailed inclusion criteria for the study participants.**


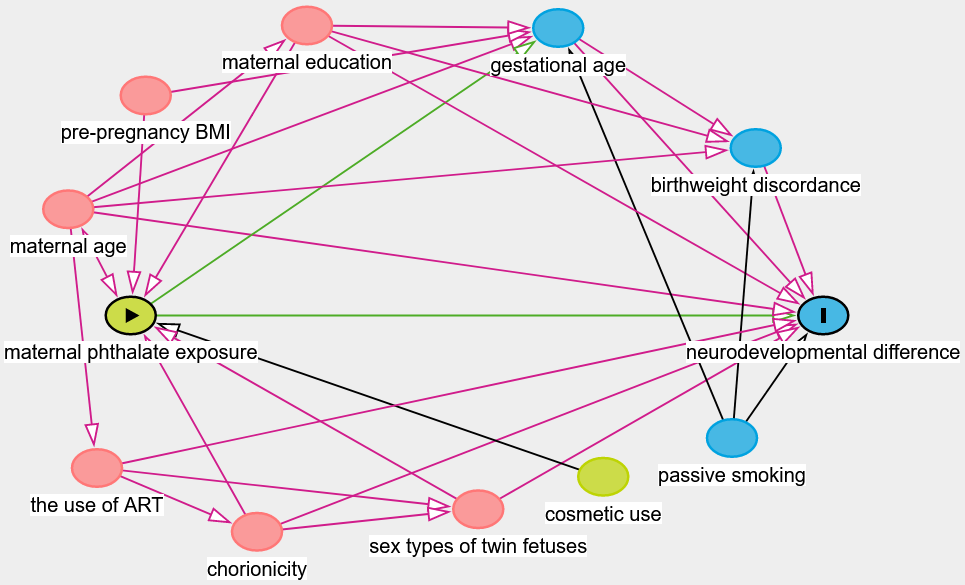

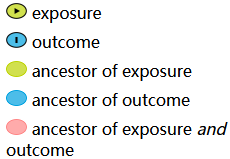


Fig. S2. **Directed acyclic graph (DAG) of the known or assumed relationships between covariates retained in the adjusted models.**

Minimal sufficient adjustment set included maternal age, pre-pregnancy BMI, education, chorionicity, and sex types of twin fetuses.

**Table S1.** Comparison of demographic characteristics between participants we followed and those lost to follow-up.

| Characteristics | Mean ± SD or n (%) | | *P*–value |
| --- | --- | --- | --- |
|  | Followed  (n = 97) | Unfollowed  (n = 189) |  |
| **Mother** |  |  |  |
| Maternal age (years) | 30.7 ± 3.9 | 30.3 ± 3.7 | 0.35 |
| Pre-pregnancy BMI (kg/m^2^) | 21.9 ± 3.1 | 21.6 ± 3.2 | 0.44 |
| Maternal education, n (%) |  |  | 0.36 |
| Less than high school | 38 (39.2) | 76 (40.2) |  |
| High school | 39 (40.2) | 62 (32.8) |  |
| College or above | 20 (20.6) | 51 (27.0) |  |
| Second-hand smoke exposure in pregnancy, n (%) |  |  | 0.34 |
| NO | 70 (72.2) | 126 (66.7) |  |
| YES | 27 (27.8) | 64 (33.3) |  |
| Cosmetic use during pregnancy, n (%) |  |  | 0.12 |
| NO | 50 (51.9) | 129 (45.1) |  |
| YES | 47 (48.5) | 157 (54.9) |  |
| **Twins** |  |  |  |
| Gestational age (weeks) | 36.7 ± 1.4 | 36.4 ± 2.1 | 0.23 |
| Chorionicity, n (%) |  |  | 0.43 |
| Monochorionic diamniotic | 31 (32.0) | 83 (29.0) |  |
| Dichorionic diamniotic | 66 (68.0) | 203 (71.0) |  |
| Birthweight discordance, n (%) |  |  | 0.67 |
| ≤ 20% | 84 (86.6) | 167 (88.4) |  |
| > 20% | 13 (13.4) | 22 (11.6) |  |
| Opposite sex of twin fetuses, n (%) |  |  | 0.40 |
| NO | 70 (62.1) | 129 (68.3) |  |
| YES | 27 (27.8) | 60 (31.7) |  |
| The use of ART, n (%) |  |  | 0.15 |
| NO | 68 (70.1) | 116 (61.4) |  |
| YES | 29 (29.9) | 73 (38.6) |  |

Abbreviations: SD, standard deviation; BMI, body mass index; ART, artificial reproductive technology.

**Table S2.** Comparison of median concentrations (interquartile range) of specific gravity-corrected urinary phthalate metabolites in the included and excluded pregnant women.

| Characteristics | Participants  included (n=97) | Participants  excluded (n=189) | *P*–value |
| --- | --- | --- | --- |
| First trimester |  |  |  |
| MEP | 5.67 (2.21, 12.0) | 5.63 (2.14, 12.6) | 0.96 |
| MiBP | 53.4 (19.5, 95.6) | 49.3 (23.27, 106) | 0.58 |
| MBP | 52.4 (21.4, 101) | 46.9 (19.1, 114) | 0.96 |
| MBzP | 0.40 (0.15, 1.56) | 0.59 (0.20, 2.16) | 0.27 |
| MEHP | 5.45 (2.62, 11.0) | 6.41 (2.84, 14.5) | 0.19 |
| MEOHP | 8.47 (4.64, 14.3) | 9.22 (5.22, 17.8) | 0.27 |
| MEHHP | 26.4 (13.9, 51.1) | 23.1 (11.4, 48.2) | 0.40 |
| MECPP | 9.96 (3.78, 15.2) | 8.58 (3.66, 20.6) | 0.74 |
| ∑DEHP | 5.67 (2.21, 12.0) | 5.63 (2.14, 12.6) | 0.43 |
| Second trimester |  |  |  |
| MEP | 7.23 (2.42, 13.1) | 5.28 (2.29, 13.4) | 0.69 |
| MiBP | 74.7 (28.8, 108) | 52.1 (21.7, 129) | 0.46 |
| MBP | 80.6 (31.5, 140) | 55.5 (20.4, 132) | 0.20 |
| MBzP | 0.53 (0.17, 2.71) | 0.49 (0.19, 2.47) | 0.99 |
| MEHP | 4.21 (1.35, 12.4) | 6.19 (3.24, 13.0) | 0.01 |
| MEOHP | 8.99 (5.16, 15.4) | 8.73 (4.67, 19.6) | 0.65 |
| MEHHP | 25.1 (13.3, 44.9) | 19.9 (11.5, 49.1) | 0.48 |
| MECPP | 9.86 (4.81, 17.4) | 9.23 (4.67, 22.2) | 0.69 |
| ∑DEHP | 7.23 (2.42, 13.1) | 5.28 (2.29, 13.4) | 0.59 |
| Third trimester |  |  |  |
| MEP | 5.88 (2.76, 19.1) | 4.84 (2.13, 10.4) | 0.03 |
| MiBP | 72.9 (31.4, 139) | 57.1 (23.7, 129) | 0.18 |
| MBP | 80.0 (27.3, 169) | 56.5 (22.0, 144) | 0.13 |
| MBzP | 0.72 (0.17, 2.78) | 0.69 (0.18, 2.28) | 0.70 |
| MEHP | 4.33 (1.80, 9.47) | 5.69 (2.28, 13.9) | 0.21 |
| MEOHP | 10.6 (6.13, 23.5) | 12.0 (6.31, 21.8) | 0.65 |
| MEHHP | 30.0 (16.2, 60.5) | 31.4 (14.1, 55.6) | 0.78 |
| MECPP | 11.7 (6.65, 29.2) | 14.7 (5.08, 29.8) | 0.99 |
| ∑DEHP | 5.88 (2.76, 19.1) | 4.84 (2.13, 10.4) | 0.71 |

*P*-values for the difference between pregnant women lost to follow-up and pregnant women who were followed for analysis were estimated using the Mann–Whitney U-test.

**Table S3.** Sensitive analysis: associations between ln-transformed urinary phthalate metabolites and intra-twin differences in neurodevelopment without adjustments of covariates (n = 97).

| Phthalate metabolites | | 1st trimester | |  | 2nd trimester | |  | 3rd trimester | |
| --- | --- | --- | --- | --- | --- | --- | --- | --- | --- |
|  | | β (95% CI) | *P*–value |  | β (95% CI) | *P*–value |  | β (95% CI) | *P*–value |
| Inter-twin MDI difference | |  |  |  |  |  |  |  |  |
| MEP | –0.52 (–1.49, 0.46) | 0.30 |  | –0.04 (–1.00, 0.92) | 0.93 |  | –0.47 (–1.30, 0.36) | 0.26 |  |
| MiBP | 0.25 (–1.15, 1.64) | 0.73 |  | 0.18 (–1.16, 1.51) | 0.80 |  | –1.38 (–2.96, 0.21) | 0.09 |  |
| MBP | –0.05 (–1.35, 1.25) | 0.94 |  | 0.04 (–1.24, 1.32) | 0.95 |  | –1.25 (–2.62, 0.11) | 0.07 |  |
| MBzP | –0.16 (–1.29, 0.97) | 0.78 |  | 0.09 (–0.87, 1.06) | 0.85 |  | 0.29 (–0.68, 1.26) | 0.56 |  |
| MEHP | 0.25 (–0.73, 1.22) | 0.62 |  | 0.29 (–0.34, 0.91) | 0.36 |  | –0.65 (–1.86, 0.55) | 0.29 |  |
| MEOHP | **1.85 (0.29, 3.41)** | **0.02** |  | 0.50 (–0.89, 1.90) | 0.48 |  | –0.65 (–2.07, 0.78) | 0.37 |  |
| MEHHP | **1.74 (0.42, 3.06)** | **0.01** |  | 0.66 (–0.65, 1.97) | 0.32 |  | –0.50 (–1.68, 0.69) | 0.41 |  |
| MECPP | 0.66 (–0.77, 2.10) | 0.37 |  | 0.23 (–0.95, 1.40) | 0.70 |  | –0.06 (–1.05, 0.93) | 0.90 |  |
| ∑DEHP | **1.91 (0.30, 3.52)** | **0.02** |  | 0.66 (–0.72, 2.04) | 0.35 |  | –0.41 (–1.50, 0.68) | 0.46 |  |
| Inter-twin PDI difference | |  |  |  |  |  |  |  |  |
| MEP | 0.23 (–1.40, 1.87) | 0.78 |  | –0.43 (–2.07, 1.20) | 0.60 |  | 0.63 (–0.71, 1.97) | 0.30 |  |
| MiBP | –0.05 (–2.48, 2.37) | 0.97 |  | –1.15 (–3.19, 0.90) | 0.27 |  | 0.95 (–2.04, 3.94) | 0.36 |  |
| MBP | 0.43 (–1.91, 2.76) | 0.72 |  | –0.68 (–2.71, 1.35) | 0.51 |  | 0.78 (–1.72, 3.28) | 0.51 |  |
| MBzP | –0.97 (–2.48, 0.53) | 0.20 |  | 0.63 (–0.80, 2.07) | 0.39 |  | 0.65 (–0.89, 2.19) | 0.41 |  |
| MEHP | 0.89 (–0.17, 1.94) | 0.10 |  | 0.20 (–0.84, 1.23) | 0.71 |  | 0.14 (–1.24, 1.52) | 0.84 |  |
| MEOHP | –0.70 (–3.68, 2.27) | 0.64 |  | –0.09 (–2.75, 2.58) | 0.95 |  | 1.25 (–1.60, 4.11) | 0.39 |  |
| MEHHP | –1.06 (–3.72, 1.59) | 0.43 |  | 0.27 (–2.24, 2.79) | 0.83 |  | 0.36 (–2.10, 2.81) | 0.78 |  |
| MECPP | –0.19 (–2.05, 1.67) | 0.84 |  | –0.46 (–2.50, 1.58) | 0.66 |  | 0.42 (–1.45, 2.29) | 0.66 |  |
| ∑DEHP | –1.04 (–3.82, 1.73) | 0.46 |  | 0.08 (–2.40, 2.55) | 0.95 |  | 0.81 (–1.32, 2.94) | 0.46 |  |

**Table S4.** Sensitive analysis: associations between ln-transformed urinary phthalate metabolites and intra-twin differences in neurodevelopment when controlled for additional potential cofounders (n = 97) ^a^.

| Phthalate metabolites | | 1st trimester | |  | 2nd trimester | |  | 3rd trimester | |
| --- | --- | --- | --- | --- | --- | --- | --- | --- | --- |
|  | | β (95% CI) | *P*–value |  | β (95% CI) | *P*–value |  | β (95% CI) | *P*–value |
| Inter-twin MDI difference | |  |  |  |  |  |  |  |  |
| MEP | –0.57 (–1.52, 0.38) | 0.24 |  | 0.13 (–0.78, 1.03) | 0.78 |  | –0.47 (–1.21, 0.27) | 0.21 |  |
| MiBP | 0.39 (–0.88, 1.65) | 0.55 |  | 0.55 (–0.69, 1.79) | 0.39 |  | –0.69 (–2.23, 0.85) | 0.38 |  |
| MBP | 0.05 (–1.23, 1.34) | 0.94 |  | 0.37 (–0.84, 1.58) | 0.55 |  | –0.50 (–1.84, 0.83) | 0.46 |  |
| MBzP | –0.41 (–1.44, 0.61) | 0.43 |  | 0.26 (–0.62, 1.14) | 0.57 |  | 0.04 (–0.93, 1.01) | 0.94 |  |
| MEHP | 0.46 (–0.49, 1.41) | 0.34 |  | 0.28 (–0.30, 0.85) | 0.34 |  | –0.46 (–1.51, 0.59) | 0.39 |  |
| **MEOHP** | **1.94 (0.49, 3.39)** | **0.01** |  | 0.35 (–0.87, 1.56) | 0.58 |  | –0.36 (–1.83, 1.11) | 0.63 |  |
| **MEHHP** | **1.56 (0.34, 2.78)** | **0.01** |  | 0.37 (–0.81, 1.55) | 0.54 |  | –0.26 (–1.44, 0.92) | 0.66 |  |
| MECPP | 0.89 (–0.49, 2.27) | 0.21 |  | 0.14 (–0.93, 1.20) | 0.80 |  | –0.03 (–1.04, 0.97) | 0.95 |  |
| **∑DEHP** | **1.84 (0.41, 3.28)** | **0.01** |  | 0.40 (–0.80, 1.60) | 0.51 |  | –0.24 (–1.44, 0.95) | 0.69 |  |
| Inter-twin PDI difference | |  |  |  |  |  |  |  |  |
| MEP | 0.17 (–1.46, 1.80) | 0.84 |  | –0.46 (–2.00, 1.07) | 0.55 |  | 1.08 (–0.13, 2.29) | 0.08 |  |
| MiBP | –0.36 (–2.4, 1.67) | 0.73 |  | –1.23 (–3.20, 0.75) | 0.22 |  | 0.83 (–1.6, 3.25) | 0.51 |  |
| MBP | 0.32 (–1.71, 2.35) | 0.76 |  | –0.72 (–2.60, 1.16) | 0.45 |  | 0.45 (–1.56, 2.46) | 0.66 |  |
| MBzP | –0.88 (–2.18, 0.41) | 0.18 |  | 0.74 (–0.61, 2.10) | 0.28 |  | 0.73 (–0.58, 2.04) | 0.27 |  |
| MEHP | 0.88 (–0.20, 1.95) | 0.11 |  | 0.22 (–0.82, 1.26) | 0.68 |  | 0.46 (–0.66, 1.58) | 0.42 |  |
| MEOHP | -1.30 (-4.29, 1.68) | 0.39 |  | 0.03 (-2.49, 2.55) | 0.98 |  | 1.79 (-0.59, 4.16) | 0.14 |  |
| MEHHP | –1.50 (–4.27, 1.27) | 0.29 |  | 0.34 (–2.11, 2.78) | 0.79 |  | 0.98 (–1.10, 3.07) | 0.36 |  |
| MECPP | –0.76 (–2.56, 1.05) | 0.41 |  | –0.66 (–2.61, 1.30) | 0.51 |  | 0.56 (–1.08, 2.21) | 0.50 |  |
| ∑DEHP | –1.61 (–4.46, 1.23) | 0.27 |  | 0.11 (–2.32, 2.54) | 0.93 |  | 1.31 (–0.54, 3.16) | 0.16 |  |

^a^ Models are adjusted for maternal age, pre-pregnancy BMI, education, chorionicity, sex types of twin fetuses, and self-reported cosmetic use.

**Table S5.** Sensitive analysis: associations between ln-transformed urinary phthalate metabolites and intra-twin differences in neurodevelopment of children conceived via natural pregnancy (n = 68).

| Phthalate metabolites | | 1st trimester | |  | 2nd trimester | |  | 3rd trimester | |
| --- | --- | --- | --- | --- | --- | --- | --- | --- | --- |
|  | | β (95% CI) | *P*–value |  | β (95% CI) | *P*–value |  | β (95% CI) | *P*–value |
| Inter-twin MDI difference | |  |  |  |  |  |  |  |  |
| MEP | –0.37 (–1.53, 0.79) | 0.53 |  | 0.02 (–0.95, 1.00) | 0.96 |  | –0.23 (–1.23, 0.77) | 0.65 |  |
| MiBP | 0.93 (–0.83, 2.69) | 0.3 |  | 0.43 (–1.1, 1.96) | 0.58 |  | –0.46 (–2.58, 1.65) | 0.67 |  |
| MBP | 0.74 (–1.16, 2.63) | 0.45 |  | 0.40 (–1.01, 1.82) | 0.58 |  | –0.12 (–2.03, 1.79) | 0.9 |  |
| MBzP | –0.08 (–1.51, 1.34) | 0.91 |  | –0.36 (–1.43, 0.71) | 0.51 |  | –0.18 (–1.35, 0.99) | 0.76 |  |
| MEHP | 0.82 (–0.67, 2.31) | 0.28 |  | 0.47 (–0.24, 1.17) | 0.19 |  | –0.64 (–1.82, 0.55) | 0.29 |  |
| MEOHP | **2.93 (0.95, 4.90)** | **0.003** |  | 0.45 (–1.21, 2.11) | 0.59 |  | –0.75 (–2.87, 1.31) | 0.49 |  |
| MEHHP | **2.37 (0.84, 3.90)** | **0.002** |  | 0.67 (–0.74, 2.07) | 0.35 |  | –0.18 (–1.80, 1.45) | 0.83 |  |
| MECPP | 0.80 (–0.8, 2.39) | 0.33 |  | 0.40 (–0.97, 1.76) | 0.57 |  | 0.01 (–1.40, 1.42) | 0.99 |  |
| ∑DEHP | **2.89 (1.01, 4.78)** | **0.003** |  | 0.71 (–0.8, 2.23) | 0.36 |  | –0.44 (–1.82, 0.94) | 0.53 |  |
| Inter-twin PDI difference | |  |  |  |  |  |  |  |  |
| MEP | 0.90 (–0.65, 2.46) | 0.25 |  | –0.04 (–1.75, 1.67) | 0.96 |  | 0.84 (–0.86, 2.53) | 0.33 |  |
| MiBP | –1.18 (–3.36, 0.99) | 0.29 |  | –1.41 (–3.94, 1.11) | 0.27 |  | 0.10 (–3.32, 3.53) | 0.95 |  |
| MBP | –1.58 (–40, 0.85) | 0.20 |  | –1.17 (–3.47, 1.14) | 0.32 |  | –0.17 (–2.95, 2.61) | 0.90 |  |
| MBzP | –0.80 (–2.33, 0.74) | 0.31 |  | 0.67 (–0.87, 2.20) | 0.39 |  | 1.48 (0.04, 2.92) | 0.04 |  |
| MEHP | 1.59 (0.05, 3.12) | 0.04 |  | 0.52 (–0.51, 1.55) | 0.32 |  | 0.54 (–0.67, 1.75) | 0.38 |  |
| MEOHP | –1.22 (–4.75, 2.32) | 0.50 |  | –1.31 (–4.21, 1.59) | 0.38 |  | 1.84 (–1.77, 5.45) | 0.32 |  |
| MEHHP | –0.47 (–3.44, 2.50) | 0.76 |  | –0.88 (–3.47, 1.71) | 0.51 |  | 1.53 (–1.29, 4.35) | 0.29 |  |
| MECPP | –0.35 (–2.43, 1.72) | 0.74 |  | –1.01 (–3.14, 1.13) | 0.36 |  | 0.54 (–1.66, 2.74) | 0.63 |  |
| ∑DEHP | –0.63 (–3.88, 2.62) | 0.70 |  | –0.74 (–3.30, 1.83) | 0.57 |  | 1.13 (–1.20, 3.46) | 0.34 |  |

^a^ Models are adjusted for maternal age, pre-pregnancy BMI, education, chorionicity, and sex types of twin fetuses.

**Table S6.** Sensitive analysis: associations between ln-transformed urinary phthalate metabolites and intra-twin differences in neurodevelopment when excluded twins discordant for birth weight (n = 84).

| Phthalate metabolites | | 1st trimester | |  | 2nd trimester | |  | 3rd trimester | |
| --- | --- | --- | --- | --- | --- | --- | --- | --- | --- |
|  | | β (95% CI) | *P*–value |  | β (95% CI) | *P*–value |  | β (95% CI) | *P*–value |
| Inter-twin MDI difference | |  |  |  |  |  |  |  |  |
| MEP | –0.50 (–1.55, 0.56) | 0.36 |  | 0.02 (–0.89, 0.92) | 0.97 |  | –0.29 (–1.08, 0.50) | 0.47 |  |
| MiBP | 0.12 (–1.09, 1.33) | 0.84 |  | 0.28 (–1.05, 1.61) | 0.68 |  | –0.58 (–2.10, 0.94) | 0.45 |  |
| MBP | 0.01 (–1.3, 1.32) | 0.99 |  | 0.21 (–1.04, 1.46) | 0.74 |  | –0.32 (–1.74, 1.11) | 0.66 |  |
| MBzP | –0.18 (–1.38, 1.02) | 0.76 |  | 0.10 (–0.80, 1.00) | 0.83 |  | 0.32 (–0.59, 1.23) | 0.49 |  |
| MEHP | 0.46 (–0.56, 1.49) | 0.38 |  | 0.34 (–0.31, 0.99) | 0.30 |  | –0.41 (–1.56, 0.74) | 0.49 |  |
| MEOHP | **1.60 (0.05, 3.15)** | **0.04** |  | 0.45 (–0.84, 1.73) | 0.50 |  | –0.27 (–1.65, 1.12) | 0.71 |  |
| MEHHP | **1.56 (0.22, 2.89)** | **0.02** |  | 0.56 (–0.73, 1.85) | 0.40 |  | –0.14 (–1.23, 0.94) | 0.80 |  |
| MECPP | 0.58 (–1.09, 2.26) | 0.50 |  | 0.45 (–0.70, 1.61) | 0.44 |  | –0.31 (–1.29, 0.66) | 0.53 |  |
| ∑DEHP | **1.85 (0.19, 3.52)** | **0.03** |  | 0.63 (–0.70, 1.97) | 0.35 |  | –0.34 (–1.41, 0.73) | 0.54 |  |
| Inter–twin PDI difference | |  |  |  |  |  |  |  |  |
| MEP | 0.78 (–0.60, 2.17) | 0.27 |  | –0.21 (–1.72, 1.29) | 0.78 |  | 1.52 (0.29, 2.75) | 0.02 |  |
| MiBP | 0.29 (–1.77, 2.35) | 0.78 |  | –1.59 (–3.58, 0.39) | 0.12 |  | 1.24 (–1.35, 3.83) | 0.35 |  |
| MBP | 1.15 (–0.73, 3.02) | 0.23 |  | –0.93 (–2.88, 1.01) | 0.35 |  | 0.78 (–1.37, 2.93) | 0.48 |  |
| MBzP | –1.06 (–2.42, 0.29) | 0.12 |  | 0.28 (–1.13, 1.70) | 0.70 |  | 0.60 (–0.85, 2.05) | 0.42 |  |
| MEHP | 0.97 (–0.11, 2.05) | 0.08 |  | 0.69 (–0.21, 1.58) | 0.13 |  | 0.35 (–0.83, 1.53) | 0.56 |  |
| MEOHP | –0.42 (–3.08, 2.24) | 0.76 |  | 0.27 (–2.07, 2.61) | 0.82 |  | 2.06 (–0.48, 4.60) | 0.11 |  |
| MEHHP | –0.53 (–3.10, 2.05) | 0.69 |  | 0.19 (–2.21, 2.60) | 0.87 |  | 1.11 (–1.08, 3.29) | 0.32 |  |
| MECPP | –1.19 (–3.31, 0.94) | 0.27 |  | –0.62 (–2.44, 1.21) | 0.51 |  | 1.19 (–0.44, 2.83) | 0.15 |  |
| ∑DEHP | –0.57 (–3.29, 2.15) | 0.68 |  | 0.25 (–2.03, 2.52) | 0.83 |  | 1.45 (–0.47, 3.37) | 0.14 |  |

^a^ Models are adjusted for maternal age, pre-pregnancy BMI, education, chorionicity, sex types of twin fetuses, and self-reported cosmetic use.

**Table S7.** Associations between ln-transformed urinary phthalate metabolites and intra-twin differences in neurodevelopment (n = 97) ^a^.

| Phthalate metabolites | 1st trimester | |  | 2nd trimester | |  | 3rd trimester | | Adj.  *P*_tri–int_ ^b^ |
| --- | --- | --- | --- | --- | --- | --- | --- | --- | --- |
|  | β (95% CI) | Adj.  *P*–value |  | β (95% CI) | Adj.  *P*–value |  | β (95% CI) | Adj.  *P*–value |  |
| Intra-twin MDI difference | |  |  |  |  |  |  |  |  |
| MEP | –0.54 (–1.48, 0.40) | 0.51 |  | 0.07 (–0.84, 0.98) | 0.89 |  | –0.50 (–1.23, 0.23) | 0.57 | 0.62 |
| MiBP | 0.38 (–0.94, 1.69) | 0.70 |  | 0.40 (–0.83, 1.62) | 0.87 |  | –0.86 (–2.35, 0.64) | 0.57 | 0.62 |
| MBP | –0.01 (–1.32, 1.29) | 0.99 |  | 0.17 (–0.98, 1.32) | 0.87 |  | –0.71 (–2.01, 0.58) | 0.57 | 0.62 |
| MBzP | –0.29 (–1.41, 0.83) | 0.70 |  | 0.14 (–0.70, 0.98) | 0.87 |  | 0.08 (–0.87, 1.04) | 0.86 | 0.87 |
| MEHP | 0.47 (–0.50, 1.45) | 0.51 |  | 0.41 (–0.19, 1.01) | 0.87 |  | –0.42 (–1.51, 0.66) | 0.57 | 0.62 |
| MEOHP | **1.91 (0.43, 3.39)** | **0.03** |  | 0.48 (–0.77, 1.73) | 0.87 |  | –0.63 (–2.03, 0.76) | 0.57 | 0.18 |
| MEHHP | **1.56 (0.33, 2.79)** | **0.03** |  | 0.53 (–0.70, 1.75) | 0.87 |  | –0.48 (–1.63, 0.67) | 0.57 | 0.18 |
| MECPP | 0.73 (–0.64, 2.11) | 0.51 |  | 0.22 (–0.84, 1.29) | 0.87 |  | –0.23 (–1.23, 0.78) | 0.74 | 0.69 |
| ∑DEHP | **1.85 (0.39, 3.31)** | **0.03** |  | 0.57 (–0.69, 1.83) | 0.87 |  | –0.53 (–1.62, 0.56) | 0.57 | 0.18 |
| Intra-twin PDI difference | |  |  |  |  |  |  |  |  |
| MEP | 0.16 (–1.54, 1.85) | 0.86 |  | –0.53 (–2.11, 1.05) | 0.92 |  | 0.93 (–0.33, 2.18) | 0.66 | 0.67 |
| MiBP | –0.44 (–2.51, 1.62) | 0.86 |  | –1.33 (–3.28, 0.62) | 0.92 |  | 0.73 (–1.79, 3.25) | 0.66 | 0.67 |
| MBP | 0.23 (–1.86, 2.33) | 0.86 |  | –0.81 (–2.70, 1.08) | 0.92 |  | 0.46 (–1.65, 2.58) | 0.67 | 0.67 |
| MBzP | –1.01 (–2.38, 0.36) | 0.68 |  | 0.58 (–0.77, 1.93) | 0.92 |  | 0.70 (–0.63, 2.04) | 0.66 | 0.67 |
| MEHP | 0.89 (–0.19, 1.98) | 0.68 |  | 0.18 (–0.86, 1.22) | 0.98 |  | 0.39 (–0.75, 1.52) | 0.66 | 0.67 |
| MEOHP | –1.15 (–4.14, 1.84) | 0.69 |  | –0.04 (–2.50, 2.43) | 0.98 |  | 1.70 (–0.81, 4.21) | 0.66 | 0.67 |
| MEHHP | –1.27 (–4.12, 1.58) | 0.69 |  | 0.19 (–2.17, 2.55) | 0.98 |  | 0.92 (–1.23, 3.07) | 0.66 | 0.67 |
| MECPP | –0.67 (–2.47, 1.13) | 0.69 |  | –0.70 (–2.59, 1.18) | 0.92 |  | 0.47 (–1.23, 2.18) | 0.66 | 0.67 |
| ∑DEHP | –1.38 (–4.32, 1.55) | 0.69 |  | 0.02 (–2.32, 2.37) | 0.98 |  | 1.10 (–0.85, 3.05) | 0.66 | 0.67 |

Abbreviations: CI, confidence interval.

^a^ Models are adjusted for maternal age, pre-pregnancy BMI, education, chorionicity, and sex types of twin fetuses.

^b^ Score test of homogeneity of effect estimates across the three trimesters.


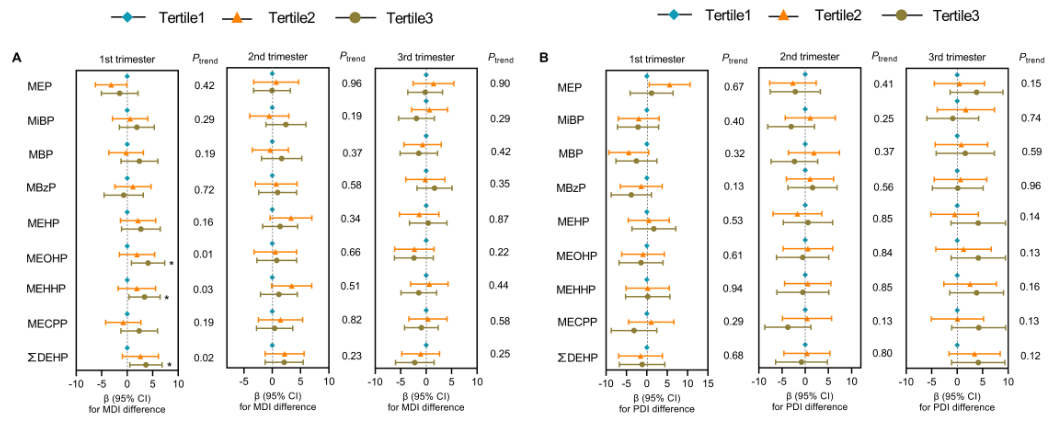


**Fig. S3.** Associations between tertiles of urinary phthalate metabolites and intra-twin difference in MDI (A) and PDI scores (B). Models are adjusted for maternal age, pre-pregnancy BMI, education, chorionicity, and sex types of twin fetuses. * *P* < 0.05


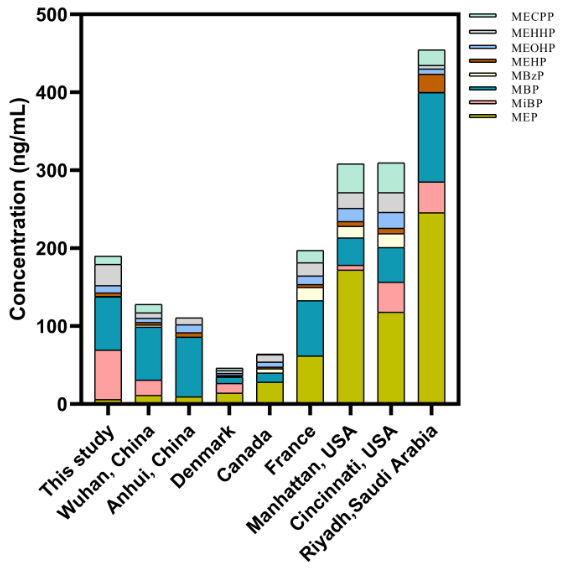


**Fig. S4.** The median concentrations of specific gravity-corrected phthalate metabolites in urine from pregnant women throughout the world. We listed the published literature below: Wuhan, China (Gao, Zhu, Xu, Zhang, Yao, Sheng et al., 2017); Anhui, China (Qian, Li, Xu, Wan, Li, Jiang et al., 2019); Demark (Bräuner, Uldbjerg, Lim, Gregersen, Krause, Frederiksen et al., 2022); Canada (Lee, Fisher, Davis, Arbuckle and Sinha, 2017); France (Jedynak, Tost, Calafat, Bourova-Flin, Broséus, Busato et al., 2022); Manhattan, USA (Doherty, Engel, Buckley, Silva, Calafat and Wolff, 2017); Cincinnati, USA (Li, Papandonatos, Calafat, Yolton, Lanphear, Chen et al., 2019); and Riyadh, Saudi Arabia (Al-Saleh, Elkhatib, Alrushud, Alnuwaysir, Alnemer, Aldhalaan et al., 2021).

**References**

Al-Saleh I, Elkhatib R, Alrushud N, Alnuwaysir H, Alnemer M, Aldhalaan H, et al. 2021. Potential health risks of maternal phthalate exposure during the first trimester - The Saudi Early Autism and Environment Study (SEAES). Environ Res 195:110882, PMID: 33621597, https://doi.org/10.1016/j.envres.2021.110882.

Bräuner EV, Uldbjerg CS, Lim YH, Gregersen LS, Krause M, Frederiksen H, et al. 2022. Presence of parabens, phenols and phthalates in paired maternal serum, urine and amniotic fluid. Environ Int 158:106987, PMID: 34991249, https://doi.org/10.1016/j.envint.2021.106987.

Doherty BT, Engel SM, Buckley JP, Silva MJ, Calafat AM, Wolff MS. 2017. Prenatal phthalate biomarker concentrations and performance on the Bayley Scales of Infant Development-II in a population of young urban children. Environ Res 152:51-58, PMID: 27741448, https://doi.org/10.1016/j.envres.2016.09.021.

Gao H, Zhu YD, Xu YY, Zhang YW, Yao HY, Sheng J, et al. 2017. Season-dependent concentrations of urinary phthalate metabolites among Chinese pregnant women: Repeated measures analysis. Environ Int 104:110-117, PMID: 28389128, https://doi.org/10.1016/j.envint.2017.03.021.

Jedynak P, Tost J, Calafat AM, Bourova-Flin E, Broséus L, Busato F, et al. 2022. Pregnancy exposure to phthalates and DNA methylation in male placenta - An epigenome-wide association study. Environ Int 160:107054, PMID: 35032864, https://doi.org/10.1016/j.envint.2021.107054.

Lee WC, Fisher M, Davis K, Arbuckle TE, Sinha SK. 2017. Identification of chemical mixtures to which Canadian pregnant women are exposed: The MIREC Study. Environ Int 99:321-330, PMID: 28040263, https://doi.org/10.1016/j.envint.2016.12.015.

Li N, Papandonatos GD, Calafat AM, Yolton K, Lanphear BP, Chen A, et al. 2019. Identifying periods of susceptibility to the impact of phthalates on children's cognitive abilities. Environ Res 172:604-614, PMID: 30878731, https://doi.org/10.1016/j.envres.2019.03.009.

Qian X, Li J, Xu S, Wan Y, Li Y, Jiang Y, et al. 2019. Prenatal exposure to phthalates and neurocognitive development in children at two years of age. Environ Int 131:105023, PMID: 31351385, https://doi.org/10.1016/j.envint.2019.105023.
